# Exposure to vehicle emissions results in altered blood brain barrier permeability and expression of matrix metalloproteinases and tight junction proteins in mice

**DOI:** 10.1186/1743-8977-10-62

**Published:** 2013-12-17

**Authors:** Hannah A Oppenheim, JoAnn Lucero, Anne-Cécile Guyot, Lindsay M Herbert, Jacob D McDonald, Aloïse Mabondzo, Amie K Lund

**Affiliations:** 1 Environmental Respiratory Health Program, Lovelace Respiratory Research Institute, Albuquerque, NM,USA; 2 CEA, Life Science Division, The Institute of Biology and Technology of Saclay, Pharmacology and Immunoanalysis Unit, Paris (Saclay), France; 3 Department of Cell Biology, University of New Mexico, Albuquerque, NM, USA; 4 Department of Biological Sciences, University of North Texas, Denton, TX, USA

**Keywords:** Air pollution, Blood brain barrier, Tight junction proteins, Matrix metalloproteinase, Neuroinflammation

## Abstract

**Background:**

Traffic-generated air pollution-exposure is associated with adverse effects in the central nervous system (CNS) in both human exposures and animal models, including neuroinflammation and neurodegeneration. While alterations in the blood brain barrier (BBB) have been implicated as a potential mechanism of air pollution-induced CNS pathologies, pathways involved have not been elucidated.

**Objectives:**

To determine whether inhalation exposure to mixed vehicle exhaust (MVE) mediates alterations in BBB permeability, activation of matrix metalloproteinases (MMP) -2 and −9, and altered tight junction (TJ) protein expression.

**Methods:**

Apolipoprotein (*Apo*) *E*^−/−^ and C57Bl6 mice were exposed to either MVE (100 μg/m^3^ PM) or filtered air (FA) for 6 hr/day for 30 days and resulting BBB permeability, expression of ROS, TJ proteins, markers of neuroinflammation, and MMP activity were assessed. Serum from study mice was applied to an *in vitro* BBB co-culture model and resulting alterations in transport and permeability were quantified.

**Results:**

MVE-exposed *Apo E*^−/−^ mice showed increased BBB permeability, elevated ROS and increased MMP-2 and −9 activity, compared to FA controls. Additionally, cerebral vessels from MVE-exposed mice expressed decreased levels of TJ proteins, occludin and claudin-5, and increased levels of inducible nitric oxide synthase (iNOS) and interleukin (IL)-1β in the parenchyma. Serum from MVE-exposed animals also resulted in increased *in vitro* BBB permeability and altered P-glycoprotein transport activity.

**Conclusions:**

These data indicate that inhalation exposure to traffic-generated air pollutants promotes increased MMP activity and degradation of TJ proteins in the cerebral vasculature, resulting in altered BBB permeability and expression of neuroinflammatory markers.

## Background

In addition to its harmful effects in the pulmonary and cardiovascular systems [1,2], several recent studies have implicated environmental air pollution-exposure in deleterious effects on the central nervous system (CNS), including neuroinflammation [3], stroke [4,5] and neurodegeneration [6]. Recent studies report a positive correlation between exposure to high levels of air pollution and increased hospital admissions/occurrence for cerebrovascular events such as stroke [4]. Air pollution-exposure has also been associated with other adverse effects on the CNS including neuroinflammation and neurodegeneration, which are associated with dementia-related disorders such as Alzheimer’s disease (AD) and Parkinson’s disease (PD) rev. in [7]. With stroke being the third leading cause of death in the Western-world, as well as the leading cause of adult disability [8,9]; and with the prevalence of neurological disorders such as AD and PD, which effect more then 4 million people in the U.S. and an estimated 27 million world-wide [10], it is critical to identify risk factors, including environmental, which may cause progression of these pathologies. While the pathways associated with air pollution-exposure induced effects on the CNS are not fully understood, recent studies suggest that pollutants, including those derived from vehicular emissions, may disrupt the integrity of the blood brain barrier (BBB) [11]. BBB disruption, and resulting alteration in permeability, has been implicated in the pathology of neurodegenerative diseases [12,13], states of neuroinflammation, rev in [13,14], and/or hemorrhagic transformation during ischemic stroke [15].

The BBB, which is comprised of endothelial cells, pericytes, and the end-foot processes of astrocytes, provides a dynamic physical and metabolic interface between the cerebral vasculature (and substances transported in the blood) and the multiple cell types found within the brain. This specialized barrier allows for specific regulation of transport into and out of the brain, in order to maintain CNS homeostasis. Between the endothelial cells that line the vascular side of the BBB are complexes that provide structural integrity, including tight junctions (TJ). TJs are continuous membrane strands that consist of three integral proteins: occludin, claudins, and junctional adhesion molecules, as well as several accessory proteins [16]. A disruption in the integrity of the BBB is often associated with decreased TJ protein expression and function. In addition to structural elements of the BBB, transport systems present at the BBB can also play a key role in maintaining CNS homeostasis [17]. One example of a CNS transporter is P-glycoprotein, which is present in several cell types in the CNS, including BBB endothelial cells, astrocytes, and microglia [18]. In the luminal BBB endothelial cells, P-glycoprotein has been shown to inhibit transport of certain toxins and drugs across the BBB into the brain, as well as regulate chemical transport from brain to blood [19].

Increased expression and activity of a family of endopeptidases, matrix metalloproteinases (MMPs), is one mechanism known to be involved in BBB disruption. MMPs are known to degrade TJ proteins, resulting in increased BBB permeability [20,21]. MMP-2 and −9, also known as gelatinases, have specifically been shown to play a significant role in BBB disruption during different pathological states [22,23].

Multiple studies now report that exposure to air pollution, including that generated from traffic-related sources, results in neurodegeneration [6,24-26] and increased expression of markers associated with neuroinflammation, including inducible nitric oxide (iNOS) and interleukin-(IL)-1β expression, all of which are associated with AD and PD-associated pathologies [27,28]. While a large portion of the more recent literature has focused on the olfactory nerve/bulb as the main site of entry and/or action of inhaled pollutants; we chose to investigate whether circulating factors present in the blood after exposure to vehicle engine-generated air pollutants may act as mediators of alterations in BBB structure and function.

We have previously reported that inhaled exposure to traffic-generated pollutants results in induction of reactive oxygen species (ROS) and MMP expression in the systemic vasculature [29,30] of *Apo E*^−/−^ mice. The *Apo E*^−/−^ mouse, when fed a Western (high fat) diet, develops atherosclerosis similar to that observed in humans. We are utilizing this model in these studies to be able to compare results observed in the systemic vasculature in previous studies with those observed in the cerebral vasculature in the current studies. Additionally, this model serves as a baseline for vascular disease that is present in most humans by adolescence. In the current studies, we tested the hypothesis that inhalation exposure to mixed gasoline and diesel vehicle emissions (MVE) results in BBB disruption that is mediated through increased expression and activity of MMPs in the cerebral vasculature, resulting in altered TJ protein expression.

## Results

### BBB Permeability is altered through exposure to MVE in *Apo E*^−/−^ mice

**Figure 1:**
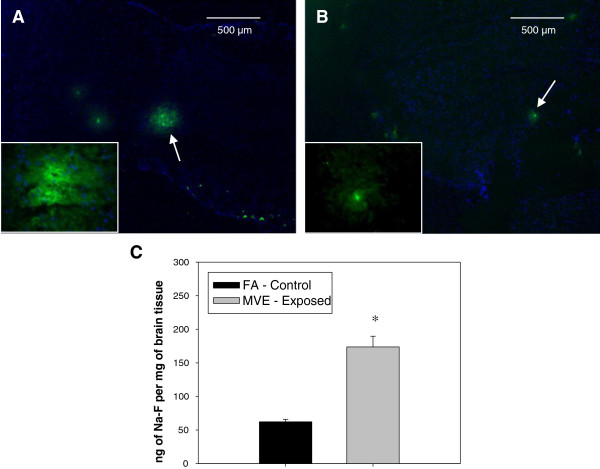
**Changes in BBB permeability in *Apo E***^**−/− **^**mice exposed to mixed vehicle emissions.** Changes in BBB permeability were assessed using the fluorescent tracer, sodium fluorescein (Na-F). *Apo E*^−/−^ mice were exposed by inhalation to either **(A)** mixed vehicular engine exhaust (MVE: 100 μg PM/m^3^) (n = 6) or **(B)** filtered air (n = 6), and were injected i.p. with 100 μl of 2% Na-F –PBS 30 min prior to the end of their final exposure on day 30. Green fluorescence (arrows) in (midsagittal) sections of cerebral cortex at 4x (40× inset) indicates increased BBB permeability; blue fluorescence is DAPI nuclear stain. Scale bar = 500 μm. **(C)** Total fluorescence in (right) half of cerebrum, as measured by fluorometry. Data is expressed as amount of tracer per gram of tissue. *p < 0.050 compared to FA control.

To determine whether inhalation exposure to MVE resulted in altered BBB permeability, *Apo E*^−/−^ mice on study where injected with the molecular tracer, Na-F, during the last 30 minutes on their final day of exposure, and resulting FITC fluorescence was quantified in the brain both by total fluorometer readings and visually by sectioning the brain and imaging it. Under normal homeostatic conditions Na-F would not be permitted to cross the BBB; however, when the BBB integrity is altered, Na-F can cross from the cerebral vessel lumen into the brain parenchyma and resulting fluorescence can be quantified. Tracer content was elevated in the brains of *Apo E*^−/−^ mice exposed to MVE (Figure 1A), compared to FA-control animals (Figure 1B). These results were confirmed through fluorescence measurements of brains (one- half of cerebrum, homogenized), which showed a nearly 3-fold increase in fluorescence of MVE-exposed vs. FA-control animals (Figure 1C). As noted in Figure 1, we do see also small amount of Na-F in the brains of *Apo E*^−/−^, which we hypothesize may be due to the altered vascular homeostasis of these animals since the Apo E protein is known to play a significant role in BBB structure [31]; however, there is a measurable increase in Na-F in MVE vs. FA exposed mice. Taken together, these results suggest that inhalation exposure to MVE disrupts BBB integrity, allowing for increased BBB permeability during (or immediately following) exposures.

### Circulating serum factors from mice exposed to MVE alter P-glycoprotein activity in BBB Co-culture

**Figure 2:**
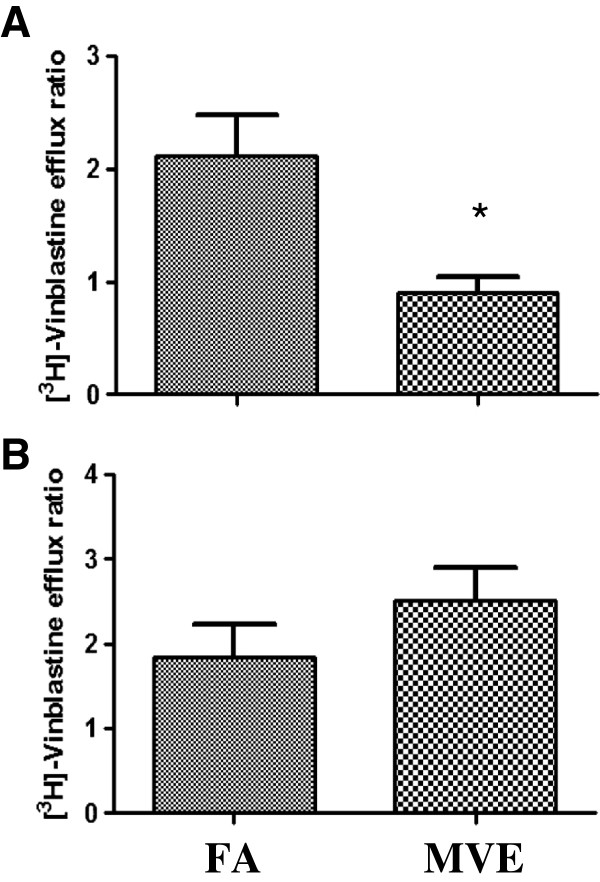
**Serum from *Apo E***^**−/− **^**mice exposed to MVE alters BBB transport activity in mouse BBB co**-**culture.** BBB function, as shown by P-glycoprotein activity, was assessed by measuring the passage of Vinblastine (0.1 μM) across BBB mouse co-cultures. Serum (1/30 dilution in media) from *Apo E*^−/−^*mice* exposed to either filtered air (FA) or mixed vehicular exhaust (MVE, 100 PM μg/m^3^), for 30 days, was applied to the apical endothelial layer transwell for **(A)** 4 hrs or **(B)** 24 hrs. [3H]-Vinblastine was then measured in both endothelial and glial well supernatants by scintillation counting at 1 hr (37°C) and resulting clearance was calculated. N = 3 per group, run in duplicate. *p < 0.050 compared to FA control.

**Figure 3:**
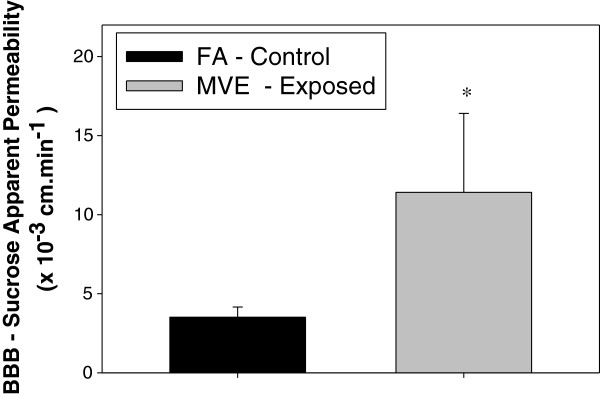
**Serum from C57Bl6 mice exposed to MVE increases BBB permeability in mouse BBB co-culture.** BBB permeability was quantified by measuring sucrose permeability across the apical membrane of the BBB co-culture. Serum (500 μl) from C57Bl6 mice exposed to either: filtered air (FA) or 100 PM μg/m^3^ of mixed vehicular emission (MVE) for 6 hr/day, for 30 days was applied to the BBB co-culture on the apical compartment (24 hrs). Data are sucrose apparent permeability coefficients (P_app_). As determined by scintillation counting (amount of tracer that passed through the apical compartment). Each data point represents the mean ± SE of n = 3 animals (three BEC monolayers each). *p < 0.050 compared to FA control.

To determine if a reactive “circulating factor” present in the blood after exposures was mediating the observed alterations in BBB permeability and function, we utilized an *in vitro* BBB model that includes a BEC (apical, transwell compartment) and glial cell (basal compartment) co-culture. Serum from MVE or FA-exposed *Apo E*^−/−^ mice was added to the apical compartment and P-glycoprotein activity was quantified by measuring the passage of Vinblastine, a P-glycoprotein substrate, across BBB mouse co-cultures. At 4 hr post-application, serum from the MVE exposure resulted in a significant decrease in P-glycoprotein transport (Figure 2A); while 24 hr after application, MVE exposure resulted in an increase in transport activity (Figure 2B). As P-glycoprotein is a major transporter that regulates entry of substances into the brain, alterations in activity suggests that exposure to MVE is mediating disruptions in BBB function in a time – dependent manner. In an effort to determine whether the circulating reactive factor, and resulting effects on the BBB, were specific to “susceptible” animals displaying underlying pathology (such as the *Apo E*^−/−^ mouse), in a separate experiment we used serum from C57Bl6 wildtype mice exposed for the same duration and concentration of either MVE or FA. Treatment of the apical compartment of the BBB co-culture with serum collected from MVE- exposed C57Bl6 mice resulted in a significant increase in BBB-permeability (Figure 3), as quantified by sucrose permeability across the membrane. These findings are in agreement with our *in vivo* results that show inhalation exposure to MVE alters BBB permeability and suggest that a factor circulating in the blood after exposure may be responsible for alterations in BBB permeability.

### MVE-Exposure results in elevated ROS in the cerebral microvasculature and parenchyma of *Apo E*^−/−^ mice

**Figure 4:**
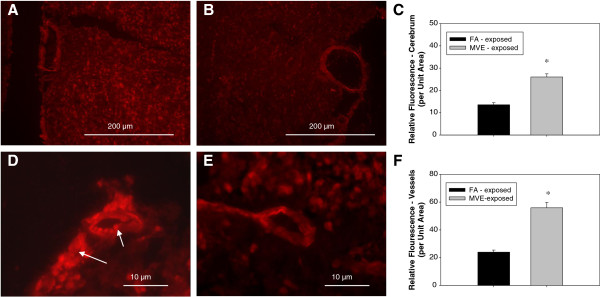
**Reactive oxygen species fluorescence staining of the cerebral cortex and cerebral microvessels from brains of *Apo E***^**−/− **^**mice exposed to mixed vehicle emissions or filtered air.** Representative dihydroethidium (ethidium, red) fluorescence staining of (frontal) cerebral cortex sections **(A,B)** and cerebral microvessels **(D,E)** from brains of *Apo E*^−/−^ mice exposed to either **(A,D)** mixed vehicular engine exhaust (MVE, 100 μg PM/m^3^) or **(B,E)** filtered air **(FA)** for 6 hr/day, 30 days. **A, B** scale bar = 100 μm; **D, E** scale bar = 10 μm. Arrows indicate areas of increased DHE staining. **C, F** = graphical quantification of total fluorescence per unit area. n = 5-6 per group, 3 slides (6 section) per animal, 2 sites (areas) each, were used for analysis. *p < 0.050 compared to FA control.

To elucidate whether exposure to MVE resulted in increased ROS levels in the cerebral vessels and parenchyma, frozen brains were analyzed for dihydroethidium staining. Ethidium fluorescence was more than 2-fold higher in nuclei in the parenchyma (Figure 4A) and nearly 3 fold higher in cerebral vessels (Figure 4D) from *Apo E*^−/−^ mice exposed to MVE for 30 days compared to that measured in those regions in FA controls (Figure 4B, 4E, respectively). Graphical representation of analysis of ethidium fluorescence is shown for both the cerebral parenchyma and cerebral microvessels in Figure 4C and 4 F.

### Exposure to MVE results in increased MMP-2 and −9 activities in the microvasculature of ***Apo E***^−/−^ mice

**Figure 5:**
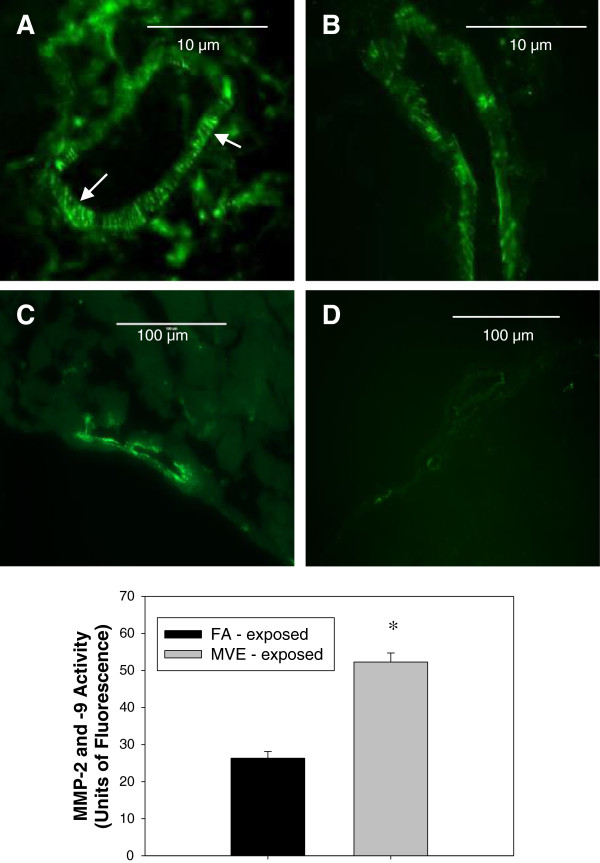
**MMP**-**9 and −2 activities in** (**frontal cortex**) **cerebral microvessels in *Apo E***^**−/− **^**mice exposed to mixed vehicle emissions or filtered air.** MMP-9 and −2 activities, as shown by *in situ* zymography, in (frontal cortex) cerebral microvessels in *Apo E*^−/−^ mice exposed to either mixed vehicle emissions (MVE: 100 μg PM/m^3^) for 6 hr/day, 30 days (**A, C**) or filtered air (FA: **B, D**). **A, B** scale bar = 10 μm; **C, D** scale bar = 100 μm. Arrows indicate increased areas of MMP activity (green fluorescence). Background fluorescence (fluorescence present in total image outside of the vessel) was subtracted from each section before statistical comparison between groups. n = 4-5 per group, 3 slides (2 sections each) per sample, 2–3 areas/locations on each sections were used for analysis. *p < 0.050 compared to FA control.

To determine if exposure to MVE altered MMP activity in cerebral microvessels of *Apo E*^−/−^ mice, we used *in situ* zymography to investigate exposure-related changes in activity of MMP-2 and −9. We observed a nearly 3-fold increase in MMP-2 and −9 activity in the cerebral microvasculature of mice exposed to MVE (Figure 5A) vs. FA controls (Figure 5B), summarized in the chart shown. Additionally, we observed a clear increase in overall MMP-2, -9 activity through the frontal lobe parenchyma in MVE-exposed (Figure 5C) animals compared to FA controls (Figure 5D).

### MVE-Exposure results in decreased expression of tight junction proteins in the cerebral microvasculature of ***Apo E***^−/−^ mice

**Figure 6:**
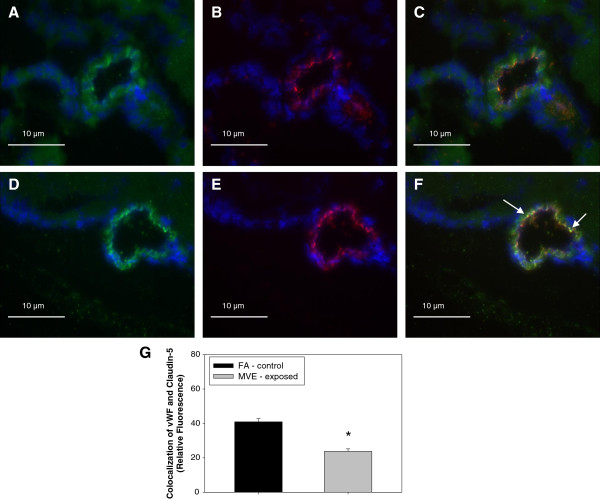
**Expression of tight junction protein claudin-5, in cerebral microvessels (frontal cortex) from *Apo E***^**−/− **^**mice exposed to mixed vehicular emission or filtered air.** Double immunofluorescence of claudin-5 (green fluorescence: **A, D**) and vonWillebrand factor (vWF) (red fluorescence: **B, E**) in cerebral microvessels (frontal cortex) from *Apo E*^−/−^ mice exposed to 100 μgPM/m^3^ of mixed vehicular emission (MVE: **A – C**) or filtered air controls (**FA**: **D - F**) for 6 hr/day, for 30 days. Colocalized expression of occludin and vWF in microvascular endothelial cells is shown in panels C and F, indicated by yellow fluorescence. Colocalization was determined by quantifying total fluorescence of overlayed signals from minimum of three slides, two sections each, three regions from each section (n = 4–5 per group), which is represented by the graph shown in panel **G**. Arrows indicate expression of claudin-5 **(D)** and endothelial-cell specific claudin-5 expression **(F)** in the cerebral microvasculature of FA animals, which is measurably decreased in the microvessels from MVE-exposed animals (**A** and **C**, respectively). Scale bar = 10 μm; 100× magnification. Control slides with no primary antibody were also done (not shown) to confirm specific binding. *p < 0.050 compared to FA control.

**Figure 7:**
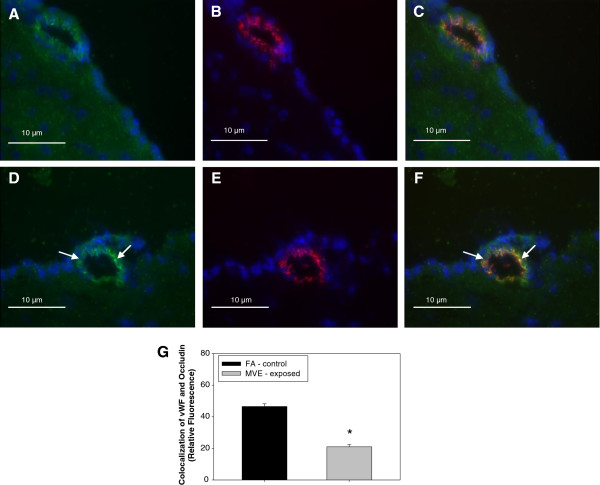
**Expression of tight junction protein occludin, in cerebral microvessels (frontal cortex) from *Apo E***^**−/− **^**mice exposed to mixed vehicular emission or filtered air.** Double immunofluorescence of occludin (green fluorescence: **A, D**) and vonWillebrand factor (vWF) (red fluorescence: **B, E**) in cerebral microvessels (frontal cortex) from *Apo E*^−/−^ mice exposed to 100 μgPM/m^3^ of mixed vehicular emission (MVE: **A – C**) or filtered air controls (**FA**: **D - F**) for 6 hr/day, for 30 days. Colocalized expression of occludin and vWF in microvascular endothelial cells is shown in panels **C** and **F**, indicated by yellow fluorescence. Colocalization was determined by quantifying total fluorescence of overlayed signals from minimum of three slides, two sections each, three regions from each section (n = 4–5 per group), which is represented by the graph shown in panel **G**. Arrows indicate expression of occludin **(D)** and endothelial-cell specific occludin expression **(F)** in the cerebral microvasculature of FA animals, which is measurably decreased in the microvessels from MVE-exposed animals (**A** and **C**, respectively). Scale bar = 10 μm; 100× magnification. Control slides with no primary antibody were also done (not shown) to confirm specific binding. *p < 0.050 compared to FA control.

**Figure 8:**
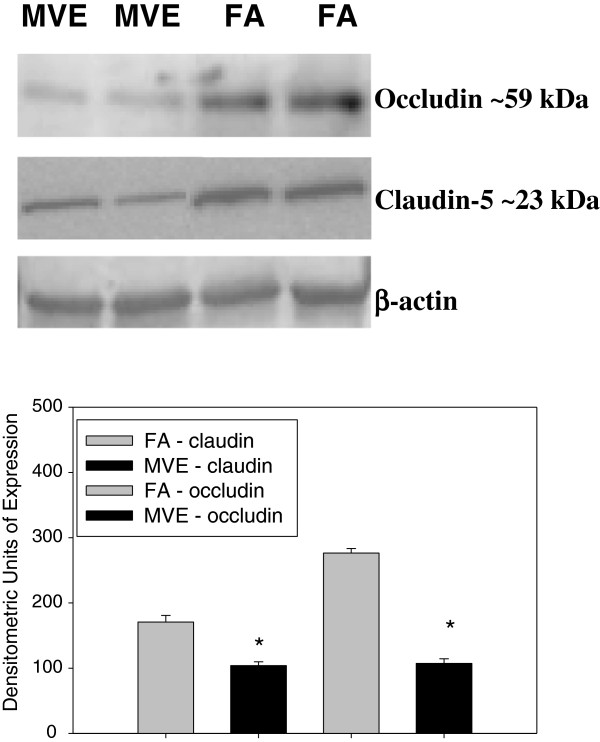
**Protein expression of claudin-5 and occludin in cerebral microvessels from *Apo E***^**−/− **^**mice exposed to either mixed vehicle emissions or filtered air.** Representative western blots of protein expression of claudin-5 and occludin from cerebral microvessels dissected from the superior aspect of the cerebrum of *Apo E*^−/−^ mice exposed to either 100 μgPM/m^3^ of mixed vehicular emission (MVE) (n = 4 pooled samples of 2 animals each) or filtered air controls (FA) (n = 4 pooled samples of 2 animals each) for 6 hr/day, for 30 days; bottom row gel – β actin loading control. Graph shows densitometric quantification of blots. *p < 0.050 compared to FA control.

In an effort to determine whether altered TJ expression may account for increased BBB permeability observed with MVE exposure, we measured the expression of TJ proteins occludin and claudin-5. Double-immunofluorescence images of cerebral vessels show a significant decrease in expression of both claudin-5 (Figure 6A) and occludin (Figure 7A) in the cerebral microvessels of MVE-exposed compared to FA-exposed (Figures 6D and 7D, respectively) *Apo E*^−/−^ mice. This decrease in expression of appears to be specific to endothelial cells present in the microvasculature as colocalized expression with vWF, an endothelial cell-specific marker, is significantly down-regulated for both claudin-5 (Figure 6C) and occludin (Figure 7C) in the MVE exposed animals, compared to FA exposed (Figures 6F and 7F, respectively), which is graphically represented in Figure 6G and Figure 7G. There is no measurable change in vWF between FA and MVE-exposed *Apo E*^−/−^ mice (Figure 6B and 6E; Figure 7B and 7E). Similar results were also observed when protein from cerebral microvessels were analyzed by Western blot (Figure 8).

### Exposure to MVE results in increased expression of markers of neuroinflammation

**Figure 9:**
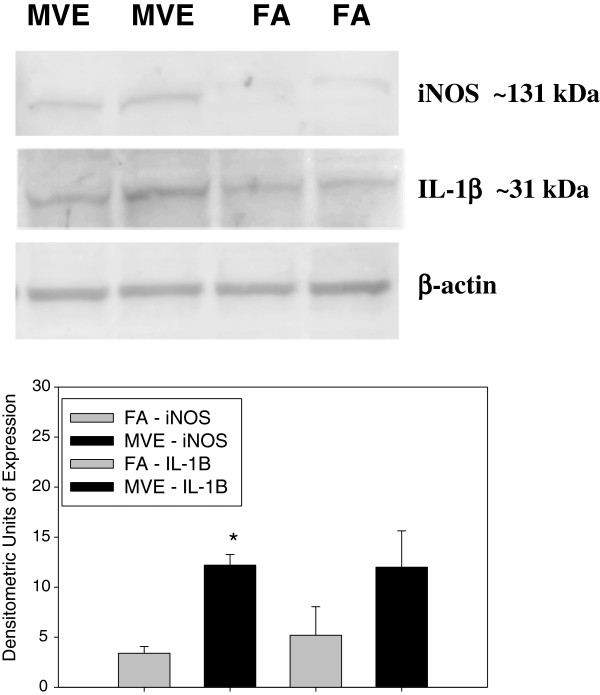
**Protein expression of inflammatory markers iNOS and IL**-**1β in cerebral parenchyma from *Apo E***^**−/− **^**mice exposed to either mixed vehicle emissions or filtered air.** Expression of iNOS and IL-1β in the cerebral parenchyma of *Apo E*^−/−^ mice exposed to either 100 μg PM/m^3^ mixed vehicle exhaust (MVE) (n = 5) or filtered air (FA) (n = 5) for 6 hr/day, for 30 days, as shown by Western blot analysis. Graph shows densitometric quantification of blots. *p < 0.050 compared to FA control.

To investigate whether the observed alterations in ROS levels, MMP activity, and expression of TJ proteins was associated with an increase in markers of neuroinflammation in the cerebral parenchyma, we measured iNOS and IL-1β, which have previously been reported to be increased in the brains of rats exposed to diesel engine exhaust [27]. The cerebrum from *Apo E*^−/−^ mice exposed to MVE show a significant increase in iNOS (Figure 9), while only a slight increase in expression of IL-1β (Figure 9) was observed, compared to FA controls.

## Discussion

There is increasing evidence that exposure to air pollutants results in harmful effects in the CNS, including increased rates of morbidity and mortality from stroke [4,5] and neurodegeneration [3,27,28]; however, the mechanisms involved are not yet fully elucidated. There are at least three proposed pathways by which components of traffic-generated air pollutants can promote effects in the brain: 1) direct transportation, via the olfactory tract; 2) via communication and signaling through the sensory afferents found in the gastrointestinal tract (brain-gut axis); and 3) through either direct transport or signaling through receptors at the BBB [11]. Here, we investigated the hypothesis that inhalation exposure to MVE results in increased BBB permeability through altered expression of MMPs and TJ protein expression, which may be mediated by a circulating factor present in the blood after exposure. Importantly, we utilized a physiologically relevant model of inhalation exposure to concentrations (100 PM μg/m^3^) of mixed diesel and gasoline engine-generated air pollutants at levels that are comparable to theoretical environmental (and occupational) scenarios. While air pollution-related effects on the CNS have been measured in young, “healthy” populations [25,28], the effects of exposure-related onset of stroke and stroke-related mortality reported in recent epidemiologic studies are primarily in adult populations with varying degrees of underlying cardiovascular disease such as atherosclerosis [32,33]. As most humans, including obese children and young adults [34], have some degree of vascular atherosclerotic plaque growth, it is important to determine whether this makes for a more “susceptible” population when exposed to environmental air pollutants. This is the primary rationale for using the atherosclerotic *Apo E*^−/−^ mouse model for the experiments presented in this manuscript. Because the Apo E protein has previously been associated with altered TJ protein expression and BBB integrity [31], it is plausible that some of the results we observed through these studies were exacerbated by the lack of Apo E protein in these mice; however, we were still able to observe statistically significant differences in the majority of our reported endpoints when comparing exposures groups.

Disruption in the structure of the BBB can result in increased permeability and a decreased ability for selective-transport from the blood to the brain. Exposure to environmental air pollutants such as tobacco smoke [35], diesel exhaust particles [26,27,36], nanoparticles [37], sulfur oxides [38], as well as chronic exposure to air pollution [28], have been associated with increased oxidative stress, neuroinflammatory signaling, and BBB disruption. We report that exposure to MVE results in altered BBB permeability in both *in vivo* and *in vitro* models, as well as altered BBB function, as shown by deregulated P-glycoprotein transport. Interestingly, we observed a significant decrease in P-glycoprotein transport at an acute time point (4 hrs) after treatment of the cells with serum from *Apo E*^−/−^ mice exposed to MVE; while 24 hr after treatment MVE exposure resulted in an increase in transport activity, albeit not statistically significant. These findings suggest that there is/are likely “circulating reactive factor(s)”, present in the serum from mice exposed to MVE, which may be responsible for promoting altered BBB permeability and function. Our *in vitro* data further suggests that there is likely a time-dependent response of exposure to air pollution and altered BBB activity. A limitation of this study is that only one time point was analyzed in the *in vitro* model, thus we are unable to clearly define a time-related response in the BBB to MVE exposure from these experiments. As serum from both C57Bl6 and *Apo E*^−/−^ mice show measurable alterations in BBB permeability and activity in our *in vitro* assays, it can be inferred that the reactive “circulating factors” resulting from MVE-exposure are likely generated in animal models with varying degrees of baseline vascular disease/pathology, as well as across different genetic backgrounds. There are multiple factors present in the circulation resulting from exposure to air pollutants that may serve as mediators of pro-inflammatory pathways and altered cell signaling and integrity at the BBB, including C-reactive protein (CRP) [39], myeloperoxidase (MPO) [40], dysfunctional high density lipoproteins (HRP) [41], and oxidized low density lipoprotein (oxLDL) [29,42]. Recent studies also show that exposure to diesel exhaust particles results in disruption of brain microvascular endothelial cells in culture, associated with increased levels of ROS [43]. Further studies are in currently in progress in our laboratory to identify which factor(s) and receptors may be mediating the observed results reported in this manuscript.

Our observation of increased levels of oxidative stress in the brain, resulting from inhalation exposure to MVE, are in agreement with other recently published findings in human populations exposed to air pollution [44]. Oxidative stress resulting from ROS is believed to play a major role in disruption of the BBB during ischemic stroke [45], in addition to altered BBB transporter function/expression and decreased TJ protein expression [26]. ROS have also been associated with activation of MMP-2 and MMP-9 in the cells of the BBB, which are known to regulate degradation of components of the basal membrane [46], resulting in disruption of TJ formation and subsequent increased BBB permeability [47]. Increased MMP activity has also been implicated in neuronal cell death and neurodegeneration [48]. Our results show that inhalation exposure to MVE results in increased ROS in both the cerebral microvasculature and parenchyma in brains of *Apo E*^−/−^ mice, which is associated with significant elevations in MMP-2 and −9 expression and activity. While we observed a more significant increase in MMP-2 and −9 activity in the cerebral vasculature, compared to the parenchyma in the brains of MVE-exposed animals, it is possible that there are spatiotemporal changes in MMP-2 or −9 expression that occur in chronic vs. acute exposures. It is also important to keep in mind that different types of air pollutants (or even different mixtures) can affect the resulting levels of ROS generated in the BBB, as the oxidative potential of environmental air pollution results from the type and concentration of its constituents (e.g. PM size/composition, volatile organic chemicals, etc.); rev in [49,50].

Increased activity of certain MMPs, such as MMP-2 and MMP-9, has been implicated in altering BBB permeability through disruption of TJ protein complexes [20]. We observed both occludin and claudin-5 expression significantly down-regulated in the cerebral microvasculature of *Apo E*^−/−^ mice exposed to MVE compared to FA, which is associated with a concomitant increase in MMP-2 and −9 activity in those vessels. Such findings suggest that MVE-initiated alterations in BBB permeability may be due, at least in part, to decreased expression of TJ proteins in the endothelial cells that constitute the BBB.

Exposure to diesel engine exhaust has been reported in the literature to result in alterations in brain function, such as hippocampal-dependent spatial learning and memory function [51], increased expression of cerebral heme oxygenase-1 (HO-1) and cyclooxygenase-2 (COX-2) [52], microglia activation [53], and neuroinflammation [27,44]. iNOS and IL-1β are common markers of neuroinflammation [54], which have been reported to be significantly up-regulated in the brain after exposure to traffic-generated air pollutants in both human exposure scenarios and animal studies [27,44]. In agreement with these studies, we observed an increase in expression of both iNOS and IL-1β expression in the cerebrum of MVE-exposed *Apo E*^−/−^ mice. While we observed a slight increase in IL-1β expression in the temporal lobe of MVE-exposed *Apo E*^−/−^ mice, measurement did not yield statistical significance in the comparison between exposure groups due to variability in baseline levels in control animals; however, it is possible IL-1β expression may be higher in other regions of the brain not analyzed in these experiments.

## Conclusions

Taken together, our results show that a 30 day inhalation exposure to MVE results in increased BBB permeability and altered BBB function observed in both *in vivo* exposures and *in vitro* models using serum from MVE-exposed animals. The findings from our preliminary *in vitro* studies suggest that a circulating factor present in the serum after the exposure may be responsible for mediating altered BBB integrity and function; however, more in depth *in vivo* studies are necessary as *in vitro* BBB co-culture models cannot directly translate to the complexity of the mammalian BBB. Furthermore, MVE-exposure results in increased levels of ROS and MMP-2 and −9 activities in the cerebral microvasculature and parenchyma, which was associated with a significant decrease in expression of TJ proteins, occludin and claudin, in *Apo E*^−/−^ mice. Consistent with previously published findings, we also observed elevations in markers of neuroinflammation, iNOS and IL-1β. While our *in vitro* results clearly suggest a role for a circulating factor (cytokine or other) in mediating alterations in BBB permeability and function after exposure to MVE, we cannot discount that results observed in our *in vivo* model of exposure (especially in regards to markers of neuroinflammation) may be due in part to other mechanisms, including direct transport of pollutants at the olfactory epithelium or via afferent signaling. Since altered BBB permeability has been implicated with increased occurrence of brain edema and hemorrhagic transformation during the acute and subacute phases of ischemic stroke [55,56], poorer prognostic stroke-related outcomes [57,58], as well as neuroinflammation and neurological pathologies, it is imperative to gain a further understanding of which environmental PM and gaseous air pollutants promote increased susceptibility and also elucidate key mechanistic pathways involved that may serve as targets for preventative therapies.

## Methods

### Animals and inhalation exposure protocol

Twelve-week-old male *Apo E*^−/−^ mice (strain B6.129P2-Apoetm1Unc N11, on a C57Bl6 background, backcrossed for 10 generations; Taconic, Oxnard, CA) were placed on a high fat diet (TD88137 Custom Research Diet, Harlan Teklad, Madison, WI; 21.2% fat content by weight, 1.5 g/kg cholesterol content) beginning 30 days prior to initiation of exposure protocol or normal rodent chow. Mice were then randomly grouped to be exposed by whole-body inhalation to a mixture of whole gasoline engine exhaust and diesel engine exhaust (MVE: 30 μg PM/m^3^ gasoline engine emissions + 70 μg PM/m^3^ diesel engine, n = 20) or filtered-air (controls, n = 20) for 6 h/d for a period of 30 days. In a separate study, 12-week old male C57Bl6 wildtype mice (Jackson Labs, Bar Harbor, Maine) fed a standard mouse chow diet, were exposed by the same methods to either filtered air (n = 8) or MVE (n = 8). MVE was created by combining exhaust from a 1996 GM gasoline engine and a Yanmar diesel generator system, as previously reported [42,59,60]. Mice were housed in standard shoebox cages within an Association for Assessment and Accreditation of Laboratory Animal Care International-approved rodent housing facility (2 m^3^ exposure chambers) for the entirety of the study, which maintained constant temperature (20–24°C) and humidity (30–60% relative humidity). Mice had access to chow and water *ad libitum* throughout the study period, except during daily exposures when chow was removed. All procedures were approved by the Lovelace Respiratory Research Institute’s Animal Care and Use Committee and conform to the *Guide for the Care and Use of Laboratory Animals* published by the US National Institutes of Health (NIH Publication No. 85–23, revised 1996).

### Tissue collection

Upon completion of the designated exposure period, animals were sacrificed 14–16 hours after their last exposure, and tissues were collected. Mice were anesthetized with Euthasol (390 mg pentobarbital sodium, 50 mg phenytoin sodium/ ml; diluted 1:10 and administered at a dose 0.1 ml per 30 g mouse) and euthanized by exsanguination. For all animals but those on the Na-F (see below) leg of the study, the brain tissue was carefully dissected from the skull, meninges were removed, and were either [1] embedded in OCT (VWR Scientific, West Chester, PA) (n = 6 FA, n = 6 MVE) and frozen on dry ice or [2] immediately snap frozen in liquid nitrogen for protein assays (n = 8 FA, n = 8 MVE). Tissue was stored at −80 ºC until assayed.

### BBB Permeability

Changes in BBB permeability were assessed using the fluorescent tracer, sodium fluorescein (Na-F) in a subset of mice on study (n = 6 MVE, n = 6 FA exposed), as previously described [61]. Briefly, *Apo E*^−/−^ mice exposed to either filtered air or mixed vehicular engine exhaust were injected intraperitoneally with 100 μl of 2% Na-F in 1x PBS 30 min prior to the end of their final exposure on day 30. Mice were anesthetized 1 hr post exposure and transcardially perfused with sterile saline until colorless perfusion was visualized. The brains were isolated, and the meninges, cerebellum, and brain stem were gently dissected away, split in half by a mid-sagittal cut and one-half of the cerebrum was embedded and frozen in OCT and sectioned at 10 μm. The other half of the cerebrum was weighed and homogenized in 10x vol of 50% TCA. The homogenate was then centrifuged at 13,000xg for 10 min at RT and the supernatant neutralized with 5 mol/L NaOH (1:0.8). Na-F fluorescence was measured at ex/em wavelengths of 440/525 nm on a fluorometer and fluorescent dye content was calculated using external standards (10 to 200 ng/ml). Data is expressed as amount of tracer per gram of tissue.

### *In situ* zymography

MMP activity was analyzed on frozen serial brain sections (10 μm thick) of the cerebrum, which were incubated with 150 μl of 10 μg/ml dye quenched (DQ)-gelatin (EnzChek, Molecular Probes, Invitrogen, Carlsbad, CA) and 1 μg/ml DAPI (nuclei stain, Invitrogen) in 1% UltraPure™ low melting point agarose (Invitrogen) cover-slipped, chilled for 5 min at 4ºC, and then incubated for 6 h in a dark, humid chamber at 37ºC. Some slides were co-incubated with a specific gelatinase inhibitor (MMP-2, -9 inhibitor IV, Chemicon, Millipore, Temecula, CA). Slides were analyzed using fluorescent microscopy and densitometry was calculated using white/black images and quantified using Image J software (NIH, Bethesda, MD; performed on 6 sections per sample, 3 regions per section, 6 samples per group). Background fluorescence (fluorescence present in total image outside of the vessel) was subtracted from each section before statistical comparison between groups.

### Double immunofluorescence

Brain sections (10 μm) were prepared for either occludin or claudin-5, and vonWillebrand factor (vWF) double immunofluorescence. Brain sections were incubated with 10% normal goat serum for 30 min at room temperature, washed in PBS, and incubated with 300 μl per section of the appropriate primary antibody (anti-rabbit or anti-sheep occludin, claudin-5: 1:500 dilution, Abcam, Cambridge, MA) and anti-goat vWF (1:1000 dilution, Abcam) diluted in rinse wash buffer [1 part 5% blocking solution (0.5 ml Normal Rabbit Serum in 10 ml 3% w/v Bovine Serum Albumin) and 4 parts Phosphate Buffered Saline (PBS)] with Hoescht nuclear stain (1 μl/ml; 300 μl/section) for 1 hr at RT. Slides were then rinsed 3 times with PBS. The slides were then incubated in 300 μl per section of a mixture of secondary antibodies Alexa Fluor 488 (anti-rabbit) and Alexa Flour 594 (anti-goat or anti-sheep) (1:1000 dilution, Vector Laboratories, Biovalley, Marne la Vallée, France) in the dark for 1 hr at room temperature. Slides were then rinsed 3 times in PBS, and cover-slipped with Aqueous Gel Mount (Sigma Aldrich, St. Louis, MO). Slides were imaged by fluorescent microscopy at 10x, 40x, and 100x using the appropriate excitation/emission filters, digitally recorded, and analyzed by image densitometry using Image J software (NIH). Double immunofluorescence was quantified by merging Alexa 488 (fluorescein isothiocyanate) and Alexa 594 (Cy3) signals into Red-Green-Blue (RGB) images. Colocalization was determined by quantifying total fluorescence of overlayed signals from minimum of three slides, two sections each, three regions from each section (n = 4 per group).

### Dihydroethidium (DHE) staining

To visualize ROS levels in the brain of study animals, sections of brains (embedded in O.C.T. and cryosectioned at 10 μm) were immediately processed through DHE staining. Slides were washed in PBS for 30 s, and rinsed 100 μl of 10 μM DHE. Slides were cover-slipped and then incubated at 37°C for 1 hr. Ethidium staining was visualized by fluorescent microscopy at 63x, digitally recorded, and analyzed by image densitometry (color images converted to white/black) using Image J software. Superoxide signal specificity was confirmed by incubating selected sections with polyethylene glycol-conjugated superoxide dismutase (PEG-SOD, 50 U/ml) for 30 min at 37°C.

### Western blot analysis

Protein levels of claudin and occludin were measured in cerebral microvessels (n = 4), and iNOS and IL-1β from the parenchyma (temporal lobe) (n = 5), from the brains of separate group of study animals via Western blot. Cerebral microvessels (arterioles and venules, targeted in the size range of less than 100 μm) were dissected from the superior surface of the cerebrum of thawed mouse brains, microscopically, on an ice-block in ice-cold HEPES-PSS. Importantly, TJ proteins claudin and occludin are heterogeneously expressed in endothelial cells of brain microvessels [62]. Vessels from 2 animals in each group were pooled for a total n value of 8 per group (n = 4 pooled samples FA, n = 4 pooled samples MVE). Protein was isolated using a RIPA buffer (50 mM Tris–HCl, pH 7.4, 150 mM NaCl, 1 mM EDTA, 1 mM PMSF, 5 μg/ml Aprotinin, 5 μg/ml Leupeptin, 1% Triton x-100, 1% Sodium deoxycholate, 0.1% SDS) for homogenization and quantified using a BCA assay (Pierce, Thermo Scientific, Rockford, IL). 5 μg of protein was loaded into each lane (n = 3–5 for each group), and subsequently run through SDS-PAGE electrophoresis under reducing conditions. After membrane transfer, membranes were blocked overnight at 4ºC in 5% blotto [5% weight/vol powdered milk: 100 ml 1X TBS (Biorad): 5% Tween vol/vol (Sigma Aldrich)]. Membranes were incubated in rabbit polyclonal anti-mouse MMP-9, claudin-5, occluding or iNOS (1:3000; Abcam), and beta-actin primary antibody (1:2000, Abcam) for 1 hour at RT. Anti-rabbit antibody conjugated to HRP (1:2000 Abcam) was used for the secondary antibody for 1 hour at RT. Bands were visualized with chemiluminescence using ECL Plus (GE Healthcare, Amersham Biosciences, Piscataway, NJ) and imaged on the FLA-5100 (Fujifilm, USA) digital image scanner; densitometry was performed utilizing Image J software (NIH).

### BBB co-culture model

Primary endothelial and glial cells from mouse were isolated and cultured as previously described [63]. Briefly, for brain endothelial cells (BEC)s, brain tissues were digested enzymatically (1 g.L^-1^ collagenase/dispase, 20 U.mL^-1^ DNAse I, 0.147 mg.L^-1^ TCLK in HBSS, 1 h at 37°C). A 20% BSA gradient was used for isolation of capillaries. After a second enzymatic digestion, cells were plated in 75-cm^2^ coated culture flasks in EBM medium completed by the EGM-2 MV SingleQuots kit (Lonza, Basel, Switzerland). Cultures were maintained at 37°C in a humidified 5% CO_2_ atmosphere for 5–6 days before being trypsinized and frozen. For BBB modelling, glial cells were seeded at a density of 5,700 cells.cm^-2^ on transwell plates in a glial-specific basal medium. BECs were plated on the upper side of a coated polyester transwell membrane (pore size 0.4 μm, Costar) in a BEC-specific medium. Microplates were then incubated at 37°C in a humidified 5% CO_2_ atmosphere for 10–12 days before treatment with serum from MVE or FA-exposed *Apo E*^−/−^ mice or C57Bl6 mice. Experiments were performed in triplicate. Upper and lower chambers will be referred to as apical and basal compartments, respectively.

### BBB permeability assay

500 μL of diluted serum (1/20) from MVE- and FA-exposed C57Bl6 mice was applied to the BBB co-culture (on the apical compartment). After 24 hr, transwells with HBMEC monolayers were transferred to new plates and a T buffer (150 mM NaCl, 5.2 mM KCl, 2.2 mM CaCl2, 0.2 mM MgCl2, 6 mM NaHCO3, 2.8 mM glucose and 5 mM Hepes) was added (1.5 ml) to the basolateral compartment and 0.5 mL to the apical compartment (A), which also contained 0.37 × 10^10^ μq/mL of [^14^C]-labeled sucrose. After 60 min incubation at 37°C, supernatants from both the A and B compartments were collected and the amount of tracer that passed through the endothelial monolayer was determined by scintillation counting. The Papp value was calculated as follows:

$$\mathrm{Papp}=\frac{\mathrm{dQ}}{\mathrm{dt}}\times A\times \mathrm{CO}$$

Where dQ/dT is the amount of compound transported per time-point, A is the membrane surface area and C0 the initial donor concentration. The mass balance (R) was calculated as:

$$R\;\left(\%\right)=100\times \left[\frac{A+D}{D0}\right]$$

Where A and D are the amounts of compounds in the acceptor and donor chambers and D0 is the amount introduced at t = 0. Mass balances of sucrose were between 80 and 120%. Monolayers were validated for sucrose permeability from A to B and B to A below 8 ×10^-6^ cm s^-1^ as reported previously [64].

### P-glycoprotein transport activity measurement

P-glycoprotein activity was quantified by measuring the passage of Vinblastine (0.1 μM), a P-glycoprotein substrate, across cell-based mouse BBB model. Serum (1/30 dilution in media) from *Apo E*^−/−^ mice exposed to either MVE or FA was applied to the apical compartment of the BBB co-culture. At 4 and 24 hr post-application of the serum, [3H]-Vinblastine was measured in both endothelial and glial well supernatants by scintillation counting at 1 hr (37°C) and resulting ratio was calculated as reported previously [63,64]. Experiments were performed in replicates of 3, two times.

### Statistical analysis

Analyses were performed using the Prism 3.0 program (GraphPad Software, Inc, San Diego, CA) for *in vitro* experiments or Sigma Stat v10 program (Systat Software, Inc, San Jose, CA) for *in vivo* experiments. Data expressed as mean ± SEM, in vitro data expressed as mean ± SD. Statistical comparisons conducted herein were accomplished using the two-tailed Student’s t-test or variance analysis (one-way ANOVA) for both *in vitro* and *in vivo* experiments. A p < 0.050 was considered statistically significant.

## Abbreviations

CNS: Central nervous system; BBB: Blood brain barrier; BECs: Brain endothelial cells; COX-2: Cyclooxygenase-2; MVE: Mixed gasoline and diesel vehicle engine emissions; TJ: Tight junction proteins; iNOS: Nitric oxide synthase; HO-1: Heme oxygenase-1; (IL)-1β: Interleukin 1-beta; MMP: Matrix metalloproteinase; AD: Alzheimer’s disease; PD: Parkinson’s disease; ROS: Reactive oxygen species; PM: Particulate matter; Apo E−/−: Apolipoprotein E knockout mouse; FA: Filtered air; Na-F: Sodium fluorescein; vWF: vonWillebrand factor; CRP: C-reactive protein; MPO: Myeloperoxidase; HDL: High density lipoprotein; oxLDL: Oxidized low density lipoprotein.

## Competing interests

The authors declare that they have no competing interests.

## Authors’ contributions

HAO participated in the design of the studies, carried out the initial histology experiments and analysis, and drafted the manuscript. JL collected study tissues, conducted histology experiments, and analyzed final data. A-CG conducted *in vitro* BBB experiments and data analysis. LMH conducted the protein analysis studies. JDM characterized and oversaw the *in vivo* animal exposures. AM participated in the study design and oversaw experiments for the in vitro BBB co-culture assays. AKL conceived of the study, participated in its design and coordination and assisted with drafting the manuscript. All authors read and approved the final manuscript prior to submission.
